# The efficiency of removing heavy metal ions from industrial electropolishing wastewater using natural materials

**DOI:** 10.1038/s41598-022-22466-9

**Published:** 2022-10-22

**Authors:** S. Charazińska, E. Burszta-Adamiak, P. Lochyński

**Affiliations:** Institute of Environmental Engineering, Wrocław University of Environmental and Life Sciences, Pl. Grunwaldzki 24, 50-365 Wrocław, Poland

**Keywords:** Pollution remediation, Environmental chemistry

## Abstract

Heavy metals are present in wastewater generated by industrial sectors, posing a threat to the environment, including surface and groundwater resources. With this in mind, there is a growing interest in finding alternative yet effective methods of removing heavy metal ions from industrial wastewater. Sorption is one of the techniques being readily applied due to the simplicity, high efficiency, production of small amounts of sludge, low investment, and the feasibility of the process over a wide range of pH and temperature. This paper deals with the treatment of industrial wastewater from electropolishing of stainless steel containing high concentrations of metal ions Fe(III), Cr(III), Ni(II), and Cu(II). Taking into account the effectiveness, availability and applicability of biosorbents for acidic wastewater, orange peels, algae, *Eclipta alba*, and eggshells were selected for the study. Sorption tests were carried out for *Eclipta alba* and the results obtained showed a best fit for the second-order kinetic model (R^2^ > 0.99) and the Langmuir isotherm model (R^2^ > 0.99). Maximum adsorption capacity was 17.92 mg/g for mixture of metal ions. The potential use of dried and calcinated eggshells was established. Both materials achieved a high removal rate of over 95%. Iron and chromium are removed from the solution first (about 100% and 90%, respectively), followed by nickel and copper ions. FT-IR and SEM with EDS measurements used to characterize materials, together with laboratory tests using real industrial effluent, made it possible to determine their mechanism of action. Specific surface area was determined for all tested materials and the values were: 1.63, 0.15 and 5.15 m^2^/g for *Eclipta alba*, dried eggshells and calcinated eggshells, respectively. The results provide grounds for optimism in the application of selected materials for industrial wastewater treatment.

## Introduction

Heavy metal pollution had become a serious environmental problem that has attracted increasing attention in recent years. Heavy metal ions are among the most undesirable of pollutants^1,2^. Many industrial sectors generate wastewater contaminated with metal ions^3–9^, which in excessive quantities can cause serious damage to the environment. One example is the electroplating industry which releases wastewater mainly from the production and processing of metal products. One of the techniques used to treat stainless steels is electropolishing^10–14^. This process is designed to change the surface properties of an object in order to improve its aesthetic value by smoothing and making the surface glossy and at the same time increasing its corrosion resistance^12,15–19^. This method has many applications including food, automotive, and medical industries^20–22^. Typical electroplating plants produce large amounts of wastewater generated during the rinsing of workpieces. These wastewaters contain large amounts of metal ions that far exceed the quality limits for industrial wastewater discharge and must therefore be treated in on-site wastewater treatment plants prior to discharge into the sewer system due to their high toxicity^23^.

The most commonly used techniques to remove toxic metal ions from polluted waters and wastewater include chemical precipitation, membrane filtration, electrocoagulation, sorption, ion exchange, etc.^24–26^. Chemical precipitation is the process of forming insoluble metal deposits by reacting a precipitating agent, often hydroxides, with dissolved metal ions^27^. This technique is currently the most widely used of treatment methods in the electroplating industry where pH adjustment to an alkaline reaction is used to maximize the precipitation of metals in the form of hydroxides in wastewater. The use of hydroxides in the precipitation process is preferred due to the availability and low cost of precipitants in most countries. The generated metal precipitates are recovered by solid separation processes such as coagulation and/or sedimentation or filtration, among others, and the metals can be extracted^28–31^. The advantages of this method are mainly its simplicity and low cost, while the disadvantages include the slow precipitation of metals, generation of excessive sludge that requires further treatment, and the long-term environmental impacts associated with sludge disposal^32^.

Contemporary research have focused on alternative methods for the removal of heavy metal ions from industrial wastewater such as electrocoagulation, adsorption using synthetic and natural adsorbents, application of magnetic field, advanced oxidation processes, membrane processes, etc.^33–35^. Among these, sorption is one of the techniques most readily used due to the simplicity of the process, cost-effectiveness, efficiency, generation of low sludge, and low reagent consumption. In addition to the fact that biodegradable natural materials can be used as sorbents, low cost is an important factor in terms of the preparation of new materials. Therefore, the use of low-cost and widely available solid materials including waste materials has emerged as a promising technique for preparing sorbents to reduce hazardous water and wastewater pollution. For this purpose, low-cost wastes such as ore materials, sewage sludge, industrial by-products, agricultural wastes, and household wastes can be used to immediately prepare sorbents or after modification^36–38^. Among them, industrial by-products, agricultural wastes, and natural materials have recently become popular in water and wastewater treatment technologies^39–41^, and the number of publications on their use to remove various pollutants is increasing. An additional benefit of using them for the industrial wastewater as well as surface water treatment in agricultural areas is the trend towards a circular economy. An example is the use of agricultural waste as biosorbents for removing nutrients from water, because the intensification of fertilization in the agricultural catchment causes eutrophication of water in this area^42^.

Although the number of literature reports on wastewater treatment with biosorbents is on the rise, the number of studies conducted on real wastewater is rather limited. Therefore, it is important to conduct studies on real systems of pollutants in order to verify the possibility of using the most promising materials. The aim of this study was to verify the effectiveness of metal ion removal using neutral-derived materials for real industrial wastewater from a stainless steel electropolishing process. Based on a previously conducted deep literature review by the author^43^, orange peels, algae, chicken eggshells, and *Eclipta alba* were selected for preliminary studies in this paper while their applicability and effectiveness for real wastewater was subsequently checked. The novelty element of the conducted research is the use of highly acidic wastewater in biosorption studies while maintaining a high removal efficiency of heavy metal ions, which is a challenge when using this method in wastewater treatment.

## Experimental procedures

### Materials

Five types of materials were used for this study. Some of them were waste products, and some were commercially available materials. Dried orange peels were obtained as a waste material from orange fruits. After grinding, they were dried at room temperature (about 20 °C) for at least 7 days until they dried up completely. They were then ground and sieved to obtain a homogeneous granular material. Orange peel powder is a commercial material with cosmetic application recommended by the manufacturer. It contains 100% *Citrus Aurantium Amara* (Orange) Peel Powder and is made in India.

Dried seaweed is ground 100% Wakame Dried Seaweed, made in China. Seaweed powder is 100% Seaweed *Ascophyllum nodosum* with origin in Norway, but produced in Poland.

*Eclipta alba* plant is 100% cut herb with origin in China. The material has been further ground before being used for further research. Another *Eclipta* used for research is the *Eclipta alba* powder made in India. *Eclipta alba* in both plant and powder form are commercially available products. Although produced in different markets abroad, they are commercially available and have been purchased in the Polish market.

As waste material, dried eggshells were obtained from a local bakery located in Wrocław (Poland). The raw eggshells were thoroughly washed and then dried for 24 h at 80 °C and then ground. The second type of eggs included in the study were dried eggshells, which in powder form (ground), were commercial material whose product composition consisted of 100% chicken eggshells with Poland as country of origin. Additionally both types of dried eggshells were subjected to a calcination process. The calcination process is described in more detail in section ‘Calcination process’.

### Industrial wastewater and chemicals

The industrial wastewater solution came from an industrial plant that deals with steel surface treatment located in Wrocław. Industrial wastewater originating from electropolishing of stainless steel, with pH 1.3, contained concentrated phosphoric acid (V) and sulphuric acid (VI) with the addition of triethanolamine. Due to the long-term operation of the process bath in industrial conditions, the initial level of wastewater contamination was rather high (ion concentration, Fe : Cr : Ni : Cu = 42.2: 12.5: 0.8: 1.3 g/kg). For the experiments conducted, wastewater with deionized (DI) water was used to obtain different initial concentrations.

For precipitation experiments and FT-IR, analytical grade reagents were also used: calcium hydroxide Ca(OH)_2_ pure p.a. (Chempur, Poland); calcium oxide CaO pure p.a. (Chempur, Poland); calcium carbonate precipitated CaCO_3_ pure p.a. (Chempur, Poland).

### Adsorption experiments

To perform all sorption tests with the industrial wastewater, 1 g of material was weighed into Falcon tubes and then 20 cm^3^ of wastewater was added. Agitation was performed at 100 rpm and constant room temperature. After all sorption tests, samples were filtered through Munktell Filter Paper No. 390. In order to carry out the ICP-OES test, the filtrate was mineralized with a 1:3 mixture of nitric acid and hydrochloric acid. Before ICP tests, the filtrate was additionally filtered with a PTFE syringe filter 0.45 μm.

To calculate the removal percentage (%R) for the initial tests, 1 g of different materials was mixed for 6 h with wastewater of total initial concentration of metal ions 625 mg/L (including 274 mg Fe/L, 133 mg Cr/L, 13 mg Cu/L, and 5 mg Ni/L). To calculate the adsorption kinetic 1 g of material and the industrial solution with a constant concentration (1000 mg Fe/L, 280 mg Cr/L, 28 mg Cu/L and 11 mg Ni/L) was used and mixed for different mixing times: 5 min, 10 min, 15 min, 30 min, 45 min, 1 h, 1.5 h, 2 h, 3 h, 4.5 h, 6 h, 15 h or 24 h. To calculate the removal percentage (%R) and adsorption capacity (q), 1 g of material was mixed for 6 h with industrial solution of different initial concentrations. The removal percentage of metal ions (%R) and adsorption capacity (q) were calculated according to Eqs. (1) and (2), respectively.

1 $$\% R = \frac{{C_{0 } - C_{e} }}{{C_{0 } }} \cdot 100\%$$

2 $$q = \frac{{\left( {C_{0 } - C_{e} } \right) \cdot V}}{W}$$

where C_0_, C_e_ are initial and final concentrations of metal ions in the solution [mg/L], V is the solution volume [L], and W is the mass of dry adsorbent [g].

### Calcination process

The calcination process was carried out in a muffle furnace. Approximately 10 g of cleaned and grounded raw eggshells were placed in a ceramic crucible and calcined in a furnace at 850 °C for 4 h. After removal from the furnace and cooling, the material was characterized by FT-IR and SEM + EDS to observe the changes that occurred due to the calcination process.

### Precipitation experiments

In order to maintain the conditions that enable repeated neutralization attempts while striving to best reflect the characteristics of the process, the selected variants were referred to the range of wastewater contamination in an industrial scale. The pH level was adjusted by adding NaOH or Ca(OH)_2_ suspension to the wastewater until the desired pH value, measured with a pH meter, was attained. During the neutralization process, the solution was continuously stirred by a magnetic stirrer. After completion of the neutralization process, the resulting solution was filtered through a Munktell Filter Paper Disc Grade 390 to separate the precipitate^23^.

To perform the precipitation experiments, 60 ml of the industrial wastewater at different initial concentrations was added to 250 ml flasks. Then, 5 g of reagents (dried eggshells, calcinated eggshells, Ca(OH)_2_, CaO, CaCO_3_) were added to the effluent solution at constant room temperature. The effluent solution with the reagent was continuously stirred with a magnetic stirrer for 3 h. After the precipitation experiments, samples were filtered through Munktell Filter Paper No. 390 to separate the precipitate from the filtrate. In order to carry out ICP-OES test, the filtrate was mineralized with a 1:3 mixture of nitric acid and hydrochloric acid. Before ICP tests, the filtrate was additionally filtered with a PTFE syringe filter 0.45 μm.

### The pH of point zero charge

The determination of the pH of point zero charge (pHpzc) of *Eclipta alba* was carried out according to the conventional method. Nine flasks filled with 50 mL of 0.1 M NaCl solution were adjusted to the initial pH of 2.0 to 10 using 0.1 M NaOH or 0.1 M HCl solutions. 0.5 g of dried *Eclipta alba* powder was added to each flask. The mixture obtained undergoes vigorous shaking to for 24 h at a speed for 150 rpm. The final pH was noted and plotted versus the initial pH, where the intersection point of the two curves determines the pHpzc of the biomass.

### Reuse potential

The determination of reusability of the materials was carried out in 4 cycles. Treatment of the industrial electopolishing wastewater with materials was carried out for 6 h with an initial total concentration of metal ions 560 ± 15 mg/L, using 50 g/L of material. Agitation was performed at 100 rpm and constant room temperature. After this process, the wastewater was separated and the material was dried. The material was then subjected to a treatment to remove metal ions from using different solutions: 0.1 M HCl, 0.1 M HNO_3_, 0.1 M NaOH and distilled water. Agitation was performed at 100 rpm and constant room temperature for 6 h. After treatment, the solution was separated, the material was rinsed with distilled water to remove the residual solution and dried. This cycle of wastewater treatment and treatment with HCl, HNO_3_, NaOH and distilled water was repeated four times. In order to carry out the ICP-OES test, all of the separated solutions were mineralized with a 1 : 3 mixture of nitric acid and hydrochloric acid. Before ICP tests, the solutions were additionally filtered with a 0.45 μm PTFE syringe filter.

### Instrumental analyses

The infrared absorption spectroscopy technique was used to measure structure of the materials. Measurements were made on a Thermo Scientific Nicolet iZ10 FT-IR (Fourier Transformation Infrared) spectrometer with a module of Thermo Scientific Nicolet iN10 MX microscope equipped with a Smart iTX accessory and a diamond plate in the area of the electromagnetic spectrum with wavelength ranging from 4000 to 400 cm^−1^. This range is most applicable to the structure of organic compounds. FT-IR spectroscopy can provide information on the broad chemical groups present in material, depending on the available absorption peaks^44^. To obtain FT-IR spectra, 32 scans were collected at the 4 cm^−1^ spectral resolution.

The scanning electron microscopy (SEM) method (Quanta 3D 200i Microscope and FEI Helios G4 PFIB CXe DualBeam Microscope) was used to investigate the surface morphology of the studied materials. The composition was analyzed by the Oxford Energy Dispersive X-ray Spectrometer (EDS) coupled to the scanning microscope. The element distribution map was obtained using the Bruker XFlash 630 EDS Detector.

ICP-OES (inductively coupled plasma–optical emission spectroscopy) tests were conducted using the Thermo Scientific iCAP 7000 Series ICP-OES apparatus with an automated sample feeder and software manufactured by Qtegra Intelligent Scientific Data Solution.

The determination of the specific surface area of solids by low-temperature nitrogen adsorption (BET) was performed using ASAP 2420 M sorption analyzer (Micromeritics) with sample degassing temperature of 200 °C.

All methods were carried out in accordance with relevant guidelines and regulations.

## Results and discussion

The selection of natural materials for the preliminary experiments was based on the conclusions of a review article published earlier by the authors^43^. Among the literature reports that treat the topic of nickel sorption with the use of natural materials, those selected had both achieved the present results and were readily available. The results of preliminary tests carried out by the authors are presented in Table 1. At the same time, attention was paid to maintaining diversity, and therefore materials belonging to different 'groups of interested researchers' were tested, such as fruit or citrus fruit fragments, algae, plant material or waste material.

**Table 1:** Characteristics of materials selected for testing.

Material	Removal of metal ions^1)^ [%]	Easiness of use	Source of origin	Commercial price^2)^ €/kg
Dried orange peel	29	High	Production waste	n/a
Orange peel powder	39	Low	Commercial	20
Dried seaweed	88	Low	Commercial	15–20
Seaweed powder	74	Low	Commercial	20
*Eclipta alba* plant	58	High	Commercial	50
*Eclipta alba* powder	99	High	Commercial	40
Dried eggshells	98	High	Production waste	n/a
Dried eggshells powder	97	High	Commercial	15
Dried eggshells + calcination	100	High	Production waste^3)^	n/a
Dried eggshells powder + calcination	100	High	Commercial^3)^	n/a

^1^Fe(III), Cr(III), Ni(II), Cu(II) from acidic electropolishing industrial wastewater. ^2^Average price in Polish market. ^3^Source of initial material before calcination process. n/a–Not available.

In the case of orange peels as both being a commercially available product and waste material, the results obtained were unsatisfactory. This material achieved a low contaminant reduction rate of less than 40%. Similarly, disappointing results were achieved by algae both in the form of larger fragments and those available commercially as powder. While the removal results were higher than for orange, the material posed problems with the application itself. Due to its nature, the dried material absorbed large amounts of solution upon contact with the wastewater solution, changing its form from dried fragments or powder to a very dense suspension. This made it very difficult to separate the material from the solution after the sorption process, and therefore the material was excluded from further testing.

Taking into account all analysed factors (Table 1), only materials characterised by a high efficiency and ease of application, i.e. *Eclipta alba* powder and dried eggshells before and after the calcination process, were selected for further studies. All of them achieved an efficiency of metal ions’ removal from the sewage solution of almost 100%, and at the same time did not exhibit the previously mentioned problems with separation of the obtained filtrate after sorption. In the case of eggshells, which came from two different sources, it was decided to use commercially available material for further studies. This was to ensure greater homogeneity of the material for further testing and to eliminate the need to obtain, clean, dry, and grind raw eggshells under laboratory conditions.

### Eclipta alba sorption kinetic and isotherms

*Eclipta alba* was one of the materials selected for a more detailed study of the sorption of metal ions from industrial electropolishing wastewater. The first step was to investigate sorption on the kinetics of the process and isotherms in order to characterize the occurring phenomena and to determine the mechanism of the process in relation to the model effluent studied.

Pseudo-first order and pseudo-second order models were then used to determine the sorption kinetics. The pseudo-second order model determined by Ho^45^ gave much better results than the pseudo-first order model proposed by Lagergren^46^. Ho's kinetic model assumes that the rate of the adsorption process is proportional to the square of the equilibrium concentration difference of the adsorbate. With the introduction of boundary conditions, this model can be transformed into a linear form, as represented by Eq. (3).

3 $$\frac{t}{{q_{t} }} = \frac{1}{{k_{2} q_{e}^{2} }} + \frac{1}{{q_{e} }} t$$

where q_e_—amount of adsorbed metal ions at equilibrium [mg/g], q_t_—amount of adsorbed metal ions at a given time t [mg/g], k_2_—rate constant of adsorption process according to pseudo-second order model [g/mg⋅min], The initial adsorption rate h [mg/(g·min)] is defined as follows:

4 $$h = k_{2} q_{e}^{2}$$

**Figure 1:**
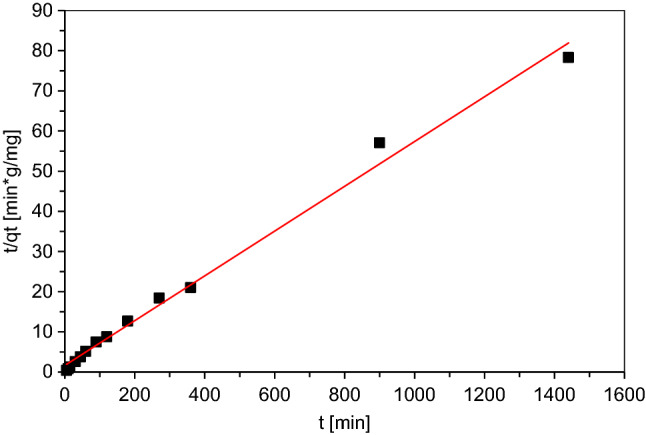
Pseudo-second order kinetic model for *Eclipta alba* powder.

The pseudo-second order model assumes that the rate of the sorption process depends on the chemical interactions resulting in the binding of metal ions on the adsorbent surface, the mechanism of complexation or ion exchange. The results for the pseudo-second order model are shown in Fig. 1, and the calculated parameters are summarized in Table 2. Summarizing the results of the adsorption kinetics study, it can be concluded that the adsorption process of metal ions (Fe, Cr, Ni, Cu) for the *Eclipta alba* powder follows the pseudo-second order kinetics model, which is consistent with the literature reports. Similar results were obtained for the *Eclipta alba* stem powder chemically modified with citric acid whose material was used for the sorption of nickel and lead ions^47^. The q_e_ value of 17.92 mg/g determined from the model is close to the value obtained by the authors in an earlier publication. Using Polish peat for sorption contaminants from the same type of electropolishing wastewater, the authors obtained value q_e_ total for all analysed metal ions (Fe, Cr, Ni and Cu) around 15 mg/g^48^. For sorption of metal ions from electroplating wastewater Sivakumar et al. used bamboo activated carbon. Sorption capacity achieved for industrial effluent containing nickel and iron was about 61.35 mg/g^49^. Salem and Awwad used modified (*Eriobotrya japonica*) loquat bark for sorption nickel ions from electroplating wastewater. According to the values obtained from the Langmuir model, the value of q_m_ was 27.548. Removal of Ni(II) by modified loquat bark from electroplating wastewater was found to be 92.4% for process parameters: concentration 12.48 mg/L Ni(II), volume 50 ml, mass of material 0.4 g. Estimated for industrial wastewater, on the basis of the presented by authors parameters and removal percentage, sorption capacity was below 2 mg/g^50^.

**Table 2:** Pseudo-second order model’s parameters for *Eclipta alba* powder.

R^2^	q_e_	k_2_	h
0.9923	17.9211	0.0019	0.6135

The authors obtained good fitting results for both pseudo-first and pseudo-second order models, with the latter obtaining a better correlation coefficient. The pseudo-second order model also obtains a better fit from other plant materials used for the sorption of metal ions from aqueous solutions, e.g. Seagrass^51^, *Q. crassipes*^52^, *Tectona grandis*^53^.

**Figure 2:**
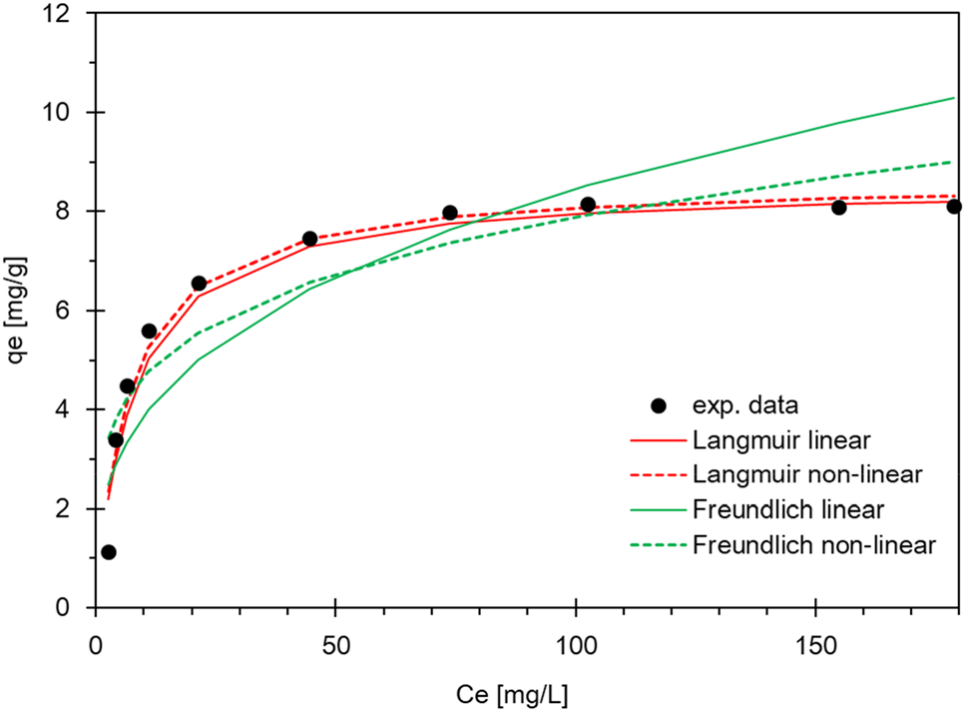
Isotherm models for biosorption onto *Eclipta alba* powder.

Isotherm modelling is very important in comparing and predicting biosorption capacity, for which two-, three-, and four-parameter models are available. Typically, two-parameter models are preferred due to ease of linearization and simplicity. The use of a more complex model is not required when two-parameter models fit the data well. Adsorption isotherms indicate the distribution of molecules between the liquid and solid phases when the adsorption process reaches equilibrium. It is used to determine the maximum sorption capacity of adsorbents and is expressed as the amount of metal adsorbed per unit mass of adsorbent used. Among the isotherm models, Langmuir^54^ and Freundlich^55^ isotherms are the most commonly used. In the present work, the two-parameter Langmuir and Freundlich models were used in linear form. The adsorption isotherms are shown in Fig. 2 and the values obtained from the analyzed models are presented in Table 3.

**Table 3:** Isotherm constants for the biosorption onto *Eclipta alba* powder.

	R^2^	q_m_	K_L_
Langmuir linear	0.9969	8.5543	0.1305
Langmuir non-linear	0.9673	8.6374	0.1422

	R^2^	n	K_f_
Freundlich linear	0.705	2.9647	1.7882
Freundlich non-linear	0.8153	2.7609	4.3859

In the Langmuir model, the surface is assumed to be homogeneous. This model clearly indicates that the adsorption sites have equal affinity for the sorbate. Moreover, adsorption at one site does not affect sorption at another. This model explains well the formation of a monolayer coverage of adsorbate on the outer surface of the adsorbent, as indicated by Eq. (4):

4 $$\frac{{C_{e} }}{{q_{e} }} = \frac{1}{{q_{m} K_{L} }} + \frac{{C_{e} }}{{q_{m} }}$$

C_e_—the metallic ion equilibrium concentration in the liquid phase [mg/L], q_e_—the equilibrium adsorption capacity [mg/g], q_m_—the adsorption isotherm constants that show the maximum adsorption capacity [mg/g], K_L_—the adsorption isotherm constants that show the adsorption energy [L/mg].

The adsorption intensity, R_L_, which is the most important feature of the Langmuir isotherm, is calculated by Eq. (5):

5 $$R_{L} = \frac{1}{{1 + K_{L} C_{i} }}$$

where C_i_ is the initial metal concentration in the liquid phase [mg/L].

The value of this parameter determines the adsorption process of nature. For 0 < R_L_ < 1, R_L_ > 1, R_L_ = 1, and R_L_ = 0, the process is reversible and desirable, non-desirable, desirable and linear, and irreversible, respectively.

The Freundlich isotherm, as an experimental model, describes the adsorption process on a heterogeneous surface. Equation (6) represents the linear form of the Freundlich isotherm model:

6 $$lnq_{e} = \frac{1}{n} lnC_{e} + lnK_{f}$$

where K_f_ [(mg/g)·(mg/L)1/n] and n are Freundlich model constants that denote the adsorption rate and degree of the adsorption process’s nonlinearity, respectively.

Out of the models tested, a better fit was obtained for the Langmuir model. The values of the correlation coefficient R^2^ for the two forms of the Langmuir model were similar with both over 0.967, but it was the linear form that proved to be the best fit to the experimental data, obtaining a very high R^2^ value of 0.997. In the case of the Freundlich model, the values were lower at about 0.705 and 0.815 for the linear and nonlinear model, respectively. From these data, the R_L_ value for the analyzed metal ions ranged from 0 to 1. This means that their adsorption was desirable and reversible. Moreover, the maximum adsorption capacity of the studied material was 8.55 and 8.64 mg/g for the linear and nonlinear model, respectively. Based on these R^2^ values, it can be concluded that the Langmuir model is able to describe the adsorption equilibrium of these ions. The values of parameter n, which are shown in Table 3, were 2.96 and 2.76, and thus in the range of n > 1, indicating that the adsorption processes taking place were physical and desirable.

The work of Ramesh Naik et al.^47^ also used the plant material *Eclipta alba* albeit chemically modified with citric acid. The equilibrium data were analyzed using Langmuir, Freundlich, Dubinin-Radushkevich, and Temkin isotherm models. It was shown that in this case, the Langmuir isotherm provides the best correlation for the biosorption of nickel and lead ions on the material.

### Eclipta alba’s efficiency in removing metal from wastewater

**Figure 3:**
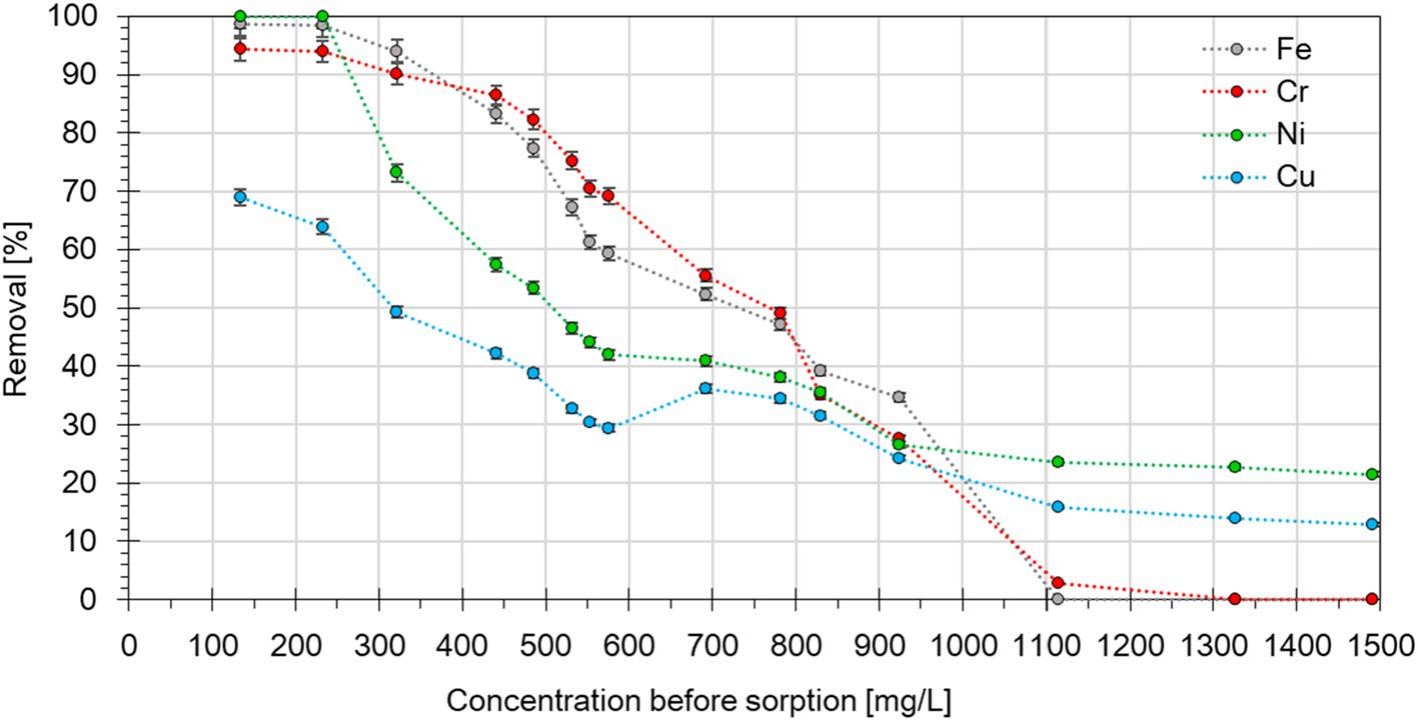
Removal percentage of metal ions from industrial solution at different initial concentrations of *Eclipta alba* powder.

By analyzing Fig. 3 on the efficiency of metal removal from wastewater, two different process characteristics can be observed with increasing concentration. The process characteristics for iron and chromium are very close to each other, while for nickel and copper they are different. Initially, at lower concentrations, almost 100% of iron and nickel and 94% of chromium are removed from the solution. Only for copper, even at low concentrations, was the maximum value less than 70%. When the initial concentration in the raw wastewater exceeds 400 mg/dm^3^, a steeper decrease in the removal efficiency of chromium and iron can be observed. For nickel, this decrease is already noticeable above 200 mg/dm^3^. For chromium and iron, with further increase in the initial concentration, a systematic decrease in the removal efficiency is observed until the limit concentration is reached. Above 1100 mg/dm^3^, the removal efficiency of iron and chromium drops practically to zero. In the case of copper and nickel, a temporary slight increase in efficiency is observed in the concentration range of about 550–900 mg/dm^3^. The process then stabilizes and the removal remains fairly stable at about 25% for nickel and about 15% for copper. A similar relationship was observed by authors in their earlier work^48^, which used peat to remove metal ions from the same type of industrial wastewater. Peat also showed similarity to the process occurring for iron and chromium but differed from nickel and copper.

The possibility of using *Eclipta prostrata* for nickel accumulation was also investigated by Chandrasekhar and Ray^56^. Experimental studies showed that this plant is moderately tolerant even to excess Ni in soil. Observations of changes in growth parameters and metal accumulation traits under the influence of different levels of Ni in soil concluded that this plant could be a metal excluder species, with potential to be used as a Ni phytostabilizer. Although Ni accumulation in roots demonstrated the plant's ability to take up Ni from the soil, growth and Ni accumulation characteristics in shoots as well as other tests showed that the plant is not a hyperaccumulating species for this metal.

### FT-IR and SEM with EDS analysis of Eclipta alba before and after sorption

Examination of the surface of the material before and after the biosorption reaction yielded information regarding the functional groups on the surface that may have participated in the process, and also indicated the sites on the surface where adsorption occurred.

**Figure 4:**
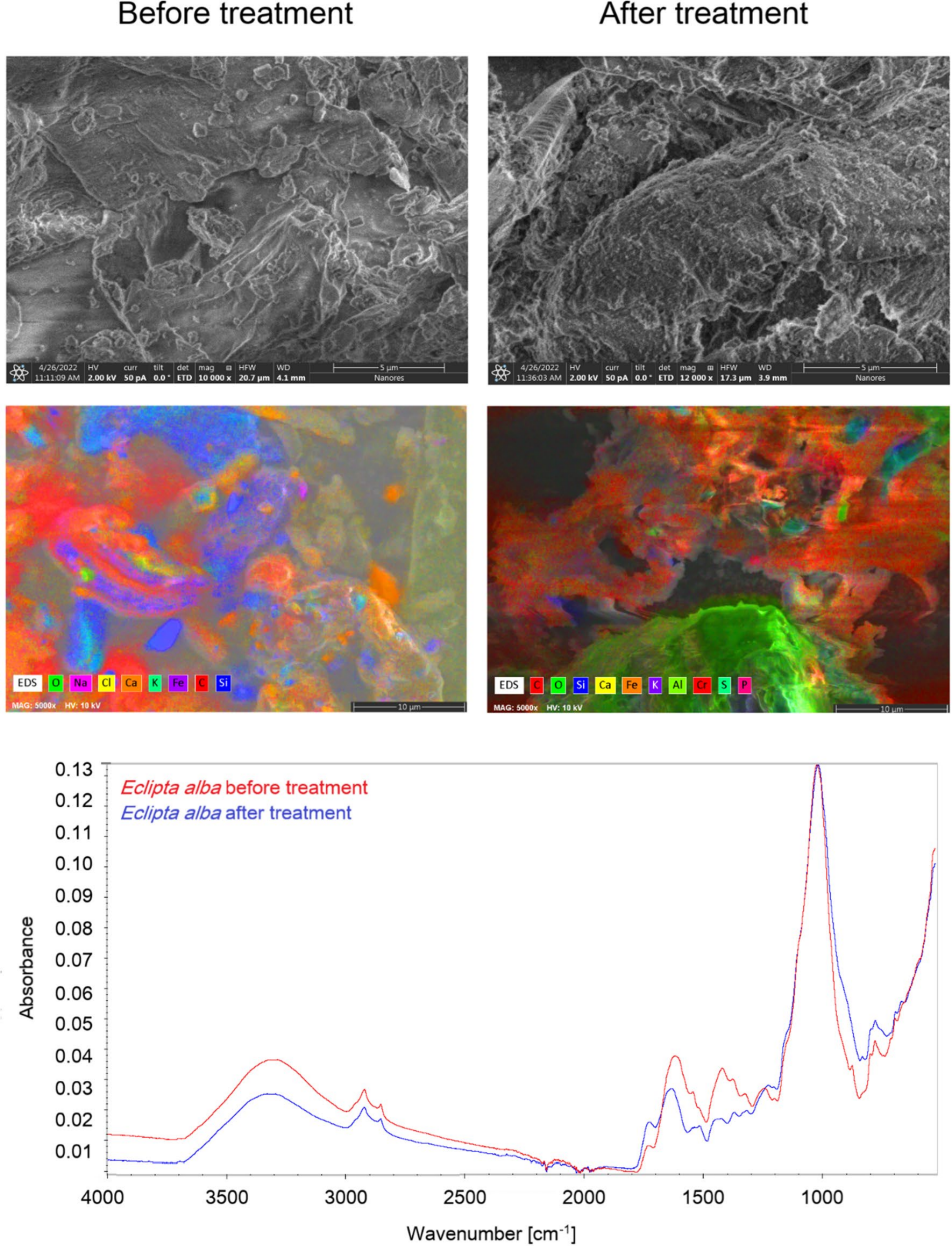
FT-IR spectra and SEM with EDS images of *Eclipta alba* powder before and after sorption.

The broad and intense absorption peaks around 3300 cm^−1^ correspond to O–H stretching vibrations of pectin, cellulose, and lignin. The peaks observed at 2900 cm^-1^ can be attributed to C–H stretching vibrations of methyl, methoxyl, and methylene groups. Symmetric stretching vibrations of ionic carboxylic groups (–COO–) appeared at 1575 cm^−1^. The C=C stretching peak was observed at 1635 cm^−1^. The bands in the range 1300–1000 cm^−1^ can be attributed to C–O stretching vibrations of alcohols and carboxylic acids. From the FT-IR spectrum of *Eclipta alba* powder, carboxyl and hydroxyl groups were present in large amounts. These groups in bio-polymers may function as proton donors, and thus deprotonated hydroxyl and carboxyl groups may be involved in coordination with metal ions. FT-IR spectra of the material after metal sorption showed changes in peak positions (Fig. 4). The peaks around 3400 cm^−1^, 1742 cm^−1^, 1635 cm^−1^, and 1575 cm^−1^ were shifted to wave numbers around 3380–3330 cm^−1^, 1755 cm^−1^, 1620 cm^−1^, and 1532 cm^−1^, respectively. These shifts can be attributed to changes in the counter ions associated with carboxylate and hydroxyl anions, suggesting that the acidic groups, carboxyl and hydroxyl, are predominantly involved in the adsorption of metal ions.

SEM analysis was used to assess the morphological changes of the adsorbent before and after the adsorption of metal ions. Figure 4 shows changes in the external morphology of the *Eclipta alba* before and after treatment. Before treatment, the surface of the material was smoother than after sorption process. The surface morphology of *Eclipta alba* after metal ions sorption become more uneven and flaky appearance.

BET analysis was measured by nitrogen adsorption isotherm, which revealed that the specific surface area of *Eclipta alba* was 1.63 m^2^/g, and pore volume for this material was 0.0082 cm^3^/g. The point zero charge determination showed that in the case of *Eclipta alba* it occurs at pH 6.2.

Desorption tests were carried out using four solutions: 0.1 M HCl, 0.1 M HNO_3_, 0.1 M NaOH and distilled water. The highest desorption efficiency was obtained for the acid solutions: 205 and 199 mg/L after the first cycle, respectively for HNO_3_ and HCl. This corresponded to 43% and 41% of the total desorbed ions, respectively. For the NaOH and distilled water solution, only 6% (27 mg/L) and 3% (15 mg/L) were obtained, respectively. After 4 cycles of desorption performed, all three of the the acid and alkaline solutions used obtained similar results of the amount of desorbed ions of about 60–70 mg/L. Again, the least effective was distilled water for which the value was 13 mg/L. Metal ion removal by *Eclipta alba* was significantly reduced even after first cycle of desorption. Regarding nickel and copper ions, the material was characterized by a lack of sorption capacity after desorption. For iron and chromium ions, only desorption with NaOH allowed the removal of these ions in the next sorption cycle, and the efficiency was about 50% for iron and 30% for chromium. Given the results obtained, the possibility of reusing *Eclipta alba* is considerably limited.

### Efficiency of metal removal from wastewater with eggshells

Eggshells are a major by-product in terms of waste generated by the egg processing industry. Eggshells have few industrial applications despite being a waste product produced in large quantities. Due to its chemical composition containing mainly calcium carbonate, several potential applications of eggshells both in their basic form and after the calcination process have been investigated. Calcined eggshells can be used as a catalyst for biodiesel production^57^, a raw material for ceramics, a fertilizer or a source of calcium in dietary supplements for animals and pets. There are also reports in the literature on the use of eggshells as a sorbent for various pollutants including phosphates, heavy metals, lignosulfonate compounds, soluble microbial products, dyes, and chlorinated organic compounds^58–61^.

Table 4 summarizes the concentrations of each metal in the raw effluent and after the neutralization process using different reactants. Five initial total concentration levels ranging from 622.8 to 3047.6 mg/L were used. For each level, the ratios of metals remained the same which for Fe, Cr, Ni, and Cu were 74.6%, 22.4%, 1%, and 2% of total concentration, respectively.

**Table 4:** Concentration of metal ions Fe(III), Cr(III), Ni(II), and Cu(II) before and after treatment.

Material	Total		Iron		Chromium		Nickel		Copper	
	before	after	before	after	before	after	before	after	before	after
	[mg/L]	[mg/L]	[mg Fe/L]	[mg Fe/L]	[mg Cr/L]	[mg Cr/L]	[mg Ni/L]	[mg Ni/L]	[mg Cu/L]	[mg Cu/L]
Calcinated eggshells	3047.6 ± 9.1	80.5 ± 2.0	2273.9	0.1	681.6	4.3	29.2	27.6	62.7	48.5
	1588.7 ± 6.3	23.9 ± 1.4	1184.3	0.3	356.0	0.2	15.2	9.0	33.2	14.4
	1016.8 ± 2.1	0.5 ± 0.1	756.9	0.1	228.3	0.0	9.9	0.0	21.6	0.4
	622.8 ± 1.8	0.6 ± 0.1	465.1	0.5	138.5	0.0	6.0	0.0	13.2	0.1
CaO	3047.6 ± 9.1	14.2 ± 0.9	2273.9	2.9	681.6	0.2	29.2	5.9	62.7	5.2
	1588.7 ± 6.3	1.9 ± 1.3	1184.3	0.5	356.0	0.1	15.2	0.6	33.2	0.8
	1016.8 ± 2.1	0.3 ± 0.1	756.9	0.1	228.3	0.1	9.9	0.0	21.6	0.1
	622.8 ± 1.8	0.2 ± 0.1	465.1	0.1	138.5	0.0	6.0	0.0	13.2	0.0
Ca(OH)_2_	3047.6 ± 9.1	7.6 ± 0.4	2273.9	2.0	681.6	0.1	29.2	1.5	62.7	4.0
	1588.7 ± 6.3	4.8 ± 1.2	1184.3	1.7	356.0	0.1	15.2	1.2	33.2	1.9
	1016.8 ± 2.1	1.5 ± 0.3	756.9	1.4	228.3	0.1	9.9	0.0	21.6	0.1
	622.8 ± 1.8	0.4 ± 0.1	465.1	0.4	138.5	0.0	6.0	0.0	13.2	0.0
Dried eggshells	3985.8 ± 10.5	188.4 ± 3.6	3012.2	9.2	859.2	70.0	35.2	34.6	74.1	62.7
	1982.4 ± 7.2	52.7 ± 1.8	1497.4	0.3	425.2	6.5	18.7	15.3	41.2	30.6
	1307.4 ± 3.1	43.0 ± 1.5	984.9	0.1	282.8	2.6	13.2	12.4	27.3	27.1
	775.28 ± 1.0	18.8 ± 1.1	583.1	0.0	168.5	0.7	7.4	6.8	16.3	11.4
CaCO_3_	3985.8 ± 10.5	95.8 ± 2.5	3012.2	0.6	859.2	0.1	35.2	33.5	62.7	61.7
	1982.4 ± 7.2	56.2 ± 1.9	1497.4	0.0	425.2	0.0	18.7	18.5	41.2	37.8
	1307.4 ± 3.1	29.5 ± 1.6	984.9	0.0	282.8	0.0	13.2	11.2	27.3	18.3
	775.28 ± 2.0	16.9 ± 1.1	583.1	0.0	168.5	0.0	7.4	6.5	16.3	10.4

Taking into account the results based on the analysis of FT-IR spectra and literature reports indicating the composition of dried eggshells and calcinated eggshells, pure chemical reagents corresponding to their composition were used to compare their effectiveness. The performance of dried eggshells was compared with calcium carbonate, while that of eggshells after calcination was compared with calcium oxide and hydroxide.

All analysed materials showed a high reduction in the total concentration of metal ions amounting to over 95%. Among the analysed metals, the highest reductions were achieved for iron, where practically 100% was removed in all cases. In the case of chromium, differences can be observed between the materials used and the chemical reagents. For calcinated eggshells, an almost complete reduction was achieved in all concentrations analysed; only for the highest concentration a chromium residue of 4.3 mg/L was observed in the purified solution. In comparison, for calcium oxide and calcium hydroxide, a reduction to less than 0.2 mg/L was achieved even for the highest concentration. A greater discrepancy between the material analyzed and the chemical reagent is seen in the case of dried eggshells. While the use of calcium carbonate allowed for a complete or nearly complete removal of chromium, for dried eggshells chromium in solution after the purification process remained for all analyzed concentration levels. However, despite these residues being in the range of 0.7–70 mg/L, removal percentage was about 92–100%. The materials used have a high efficiency of removing even very high concentrations of iron and chromium from wastewater.

Larger differences can be observed for the remaining metal ions, i.e. nickel and copper. Dried eggshells and calcium carbonate achieved similar reduction results of less than 30% for these metal ions. The nickel concentrations in the effluent solution after treatment for dried eggshells and calcium carbonate were in the ranges of approximately 34 mg/L, 15–18 mg/L, 11–12 mg/L, and 7 mg/L for successively lower initial contaminant levels. In the case of copper, differences between the two materials were somewhat more pronounced, but despite the differences the results achieved for the two materials were also similar. For calcinated eggshells and calcium monoxide and hydroxide, the results showed differences in performance. For the lower starting concentrations (622 and 1017 mg/L), an almost complete reduction of both nickel and copper was achieved with these three materials. Only at higher starting concentrations was the difference between the materials more apparent. The best performing of the three materials was calcium hydroxide, for which post-treatment concentrations of 1.5–1.2 mg/L and 4.0–1.9 mg/L for nickel and copper were achieved, respectively. For calcium oxide, the same starting concentrations 5.9–0.6 mg/L and 5.2–0.8 mg/L were achieved for nickel and copper, respectively. In the case of calcinated eggshells, these values were several times higher which for nickel were 27.6–9.0 mg/L and for copper 48.5–14.4 mg/L for the same initial concentrations.

### FT-IR and SEM with EDS analysis of eggshells before and after sorption

One of the properties affecting the removal efficiency of contaminants by the eggshells is the presence of selected functional groups in the tested material. An FT-IR analysis was carried out to characterize dried eggshells with comparation to calcium carbonate, and eggshells after calcination was compared with calcium oxide and hydroxide.

**Figure 5:**
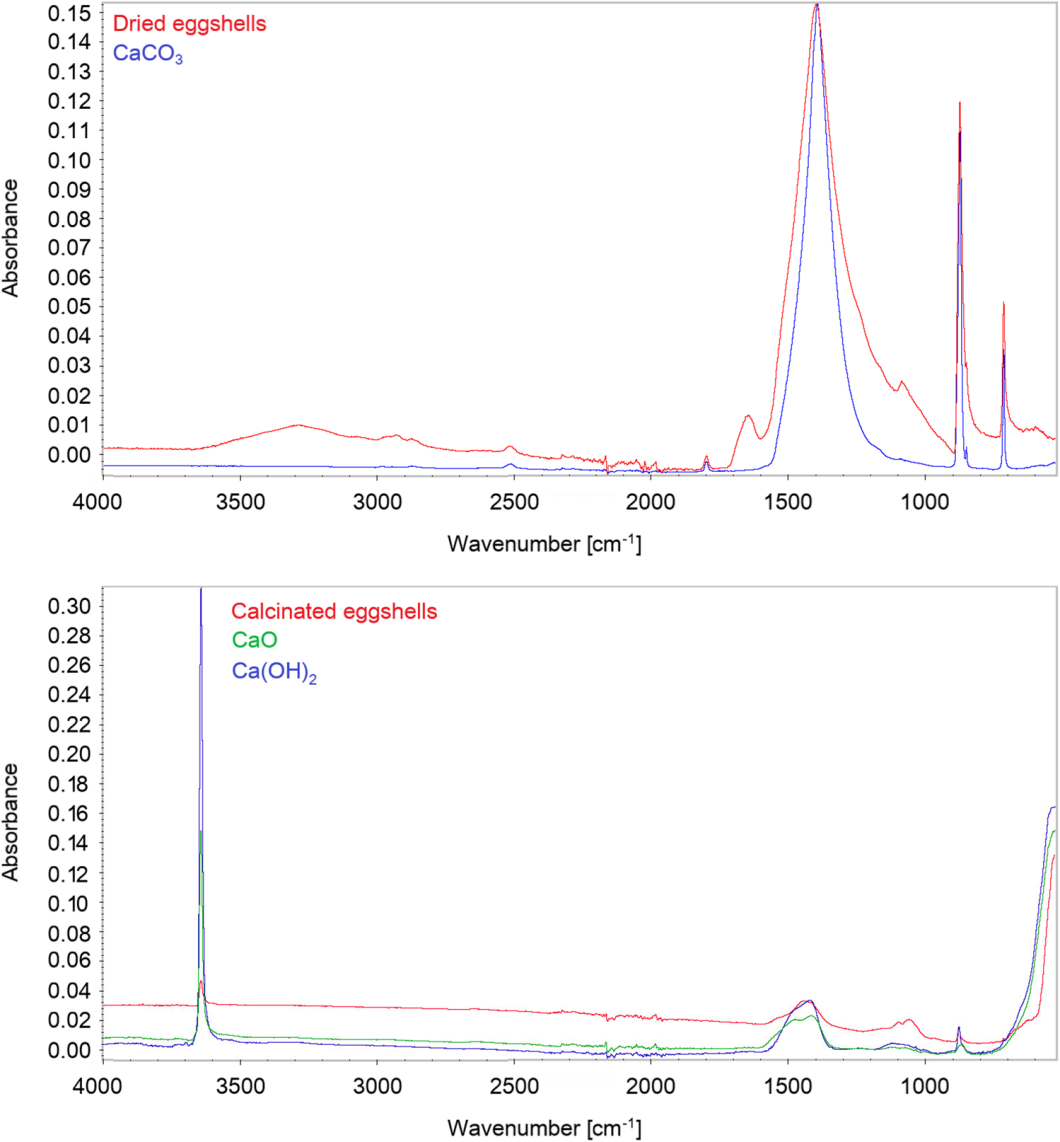
FT-IR spectra of dried eggshells and calcinated eggshells in comparison to CaCO_3,_ Ca(OH)_2_ and CaO.

Figure 5 shows the spectra of dried eggshells before and after the calcination process. The spectra obtained for the material before the calcination process correspond to the spectrum of pure calcium carbonate. In both cases, the same characteristic peaks can be observed. The broad band centering at 1394 cm^−1^ is characteristic of the bond C–O in the carbonate due to a stretching vibration, indicating a coordination bond between oxygen atoms in the carbonate and calcium atoms. Additionally, two sharp peaks can be observed at 872 and 712 cm^−1^, out-of-plane and in-plane deformation modes of carbonate, respectively. The bending vibration (scissoring) in primary amines (N–H) results in the band at 1645 cm^−1^. In the case of eggshells after the calcination process, one can observe a decrease in carbonate peaks and the appearance of a new sharp peak at 3640 cm^−1^. This peak may indicate O–H stretching vibration of Ca(OH)_2_ or CaO. This is confirmed by the combined spectra for pure Ca(OH)_2_ and CaO in which this peak is also very prominent at the same location. The obtained results indicate that the eggshells before the calcination process consist mainly of calcium carbonate, while the process itself causes the release of carbon dioxide during the process, leaving mainly CaO as the main product of the conducted process. This is also confirmed in the literature. Tangboriboon et al.^62^ have determined that eggshells before the calcination process mainly contain calcium carbonate, accounting for more than 96% of the composition, while after the calcination process 99% of the material composition was calcium oxide.

Zulfikar et al.^58^ described the process that occurs in eggshells. When eggshell powder is mixed with the solution, calcium salts can partially dissolve and release Ca^2+^ , HCO_3_^−^, CO_3_^2−^, and OH^-^ ions according to the following reaction. They also found that the resulting ions are adsorbed onto the surfaces of the eggshell particles to form a negative charge.

$${\text{CaCO}}_{{3}} + {\text{H}}_{{2}} {\text{O}} \rightleftharpoons {\text{Ca}}^{{{2} + }} + {\text{HCO}}_{3}^{ - } + {\text{OH}}^{ - } + {\text{CO}}_{3}^{2 - }$$

Vijayaraghavan et al.^63^ have also determined the process occurring between eggshells and lead ions. Given the presence of lead ions in bivalent form, the authors determined that carbonate ions from eggshells combine with Pb^2+^ to form lead carbonates. These are then adsorbed onto the surface of the material.

The literature reports are inconclusive when it comes to identifying the process responsible for the removal of metal ions from solutions using powdered or calcined eggshells. Attributing the high efficiency of the whole process only to the sorption phenomena is inconclusive, which the authors proved on the basis of comparative studies (Table 4). It should also be taken into account that sorption capacity values determined by researchers for eggshells may be overestimated. Often the authors, in calculating their effectiveness, take into account only the ion concentrations in the solution before and after the application of eggshells without allowing for the fact that some of the contaminants can be removed in the form of precipitate by precipitation and not only sorbed by the material.

**Figure 6:**
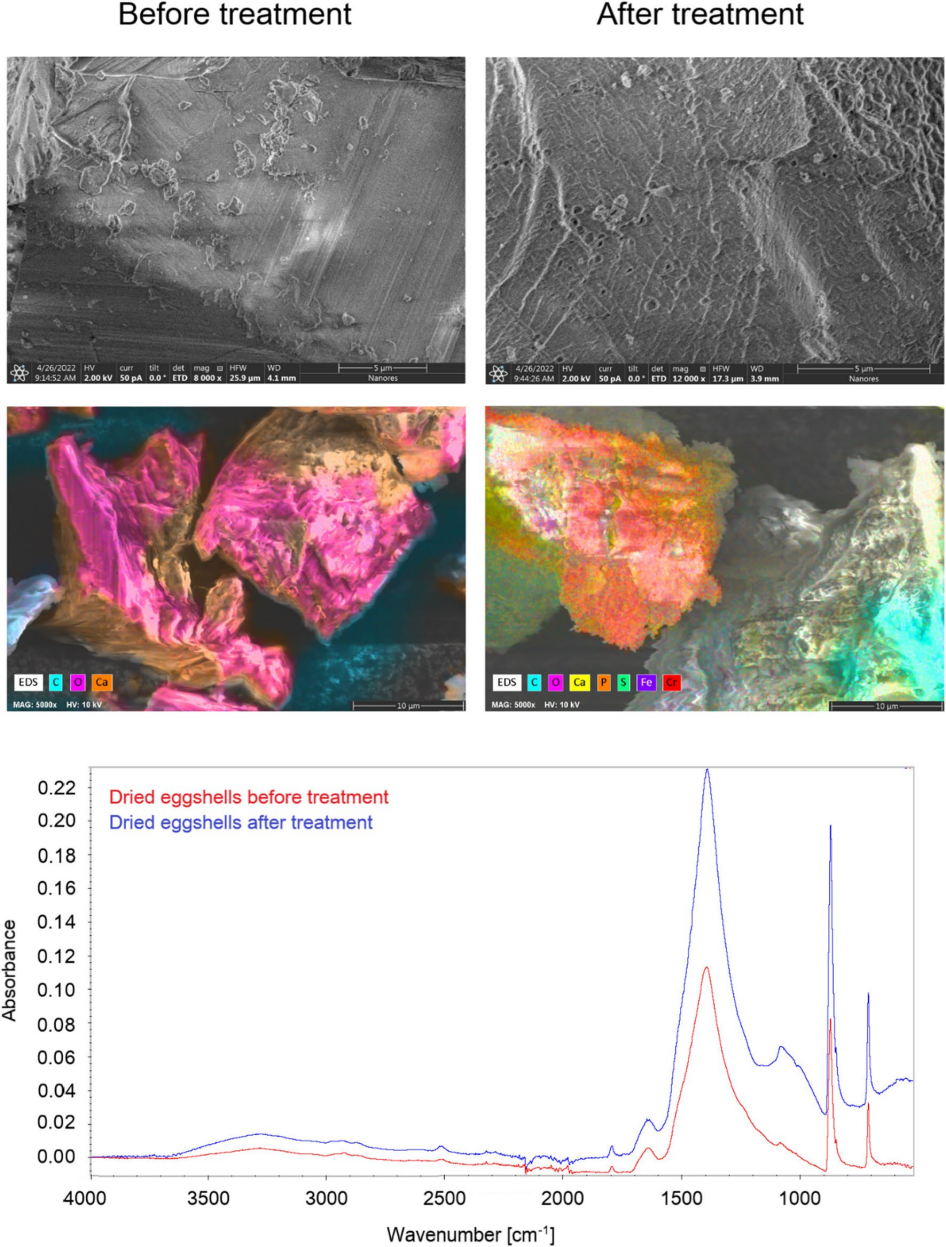
FT-IR spectra and SEM with EDS images of dried eggshells before and after sorption.

After wastewater treatment, small irregularities visible on SEM images appear on the surface. EDS analysis showed that impurities originating from sulfuric and orthophosphoric acid present in the wastewater composition, as well as iron and chromium, were retained on the material (Fig. 6).

In order to increase the efficiency of metal ion removal from wastewater, dried eggshells can be subjected to a calcination process. Calcination of eggshells at 850 °C changes the CaCO_3_ present in them into CaO. The structure visible on the SEM images of calcined eggshells differs significantly from the structure of material not subjected to this process. Calcinated eggshells consist mainly of carbon and oxygen, which is reflected in the reaction describing the processes occurring during calcination proposed by Guru and Dash^64^.

$${\text{CaCO}}_{{3}} \to {\text{CaO}} + {\text{CO}}_{{2}}$$

$${\text{CaO}} + {\text{H}}_{{2}} {\text{O}} \to {\text{Ca}}\left( {{\text{OH}}} \right)_{{2}}$$

Sankaran et al.^59^ investigated the sorption of zinc ions using calcined eggshells and found that the ions were adsorbed on the surface of the material due to electrostatic interactions and/or a cation exchange process. Kristianto et al.^65^ conducted studies with both dried eggshells and eggshells after calcination. They determined that calcination increased the sorption capacity of the material up to 60 times compared to uncalcined ones, which was observed using Langmuir and Dubinin-Radushkevich isotherms. Additionally, they determined that this process was exothermic and physisorptive. The best fit for the data was obtained for the Langmuir isotherm model which indicated monolayer adsorption onto the homogeneous surface from the adsorbent.

Another puzzling fact is the very high sorption capacity of calcinated eggshells reported by researchers, reaching up to 700 mg/g which is a rare value compared to other natural materials^43^. In order to solve the mechanism of action of calcinated eggshells, both the study of the material itself (by SEM with EDS and FT-IR) and assessing the efficiency of removing metal ions from real wastewater were carried out.

**Figure 7:**
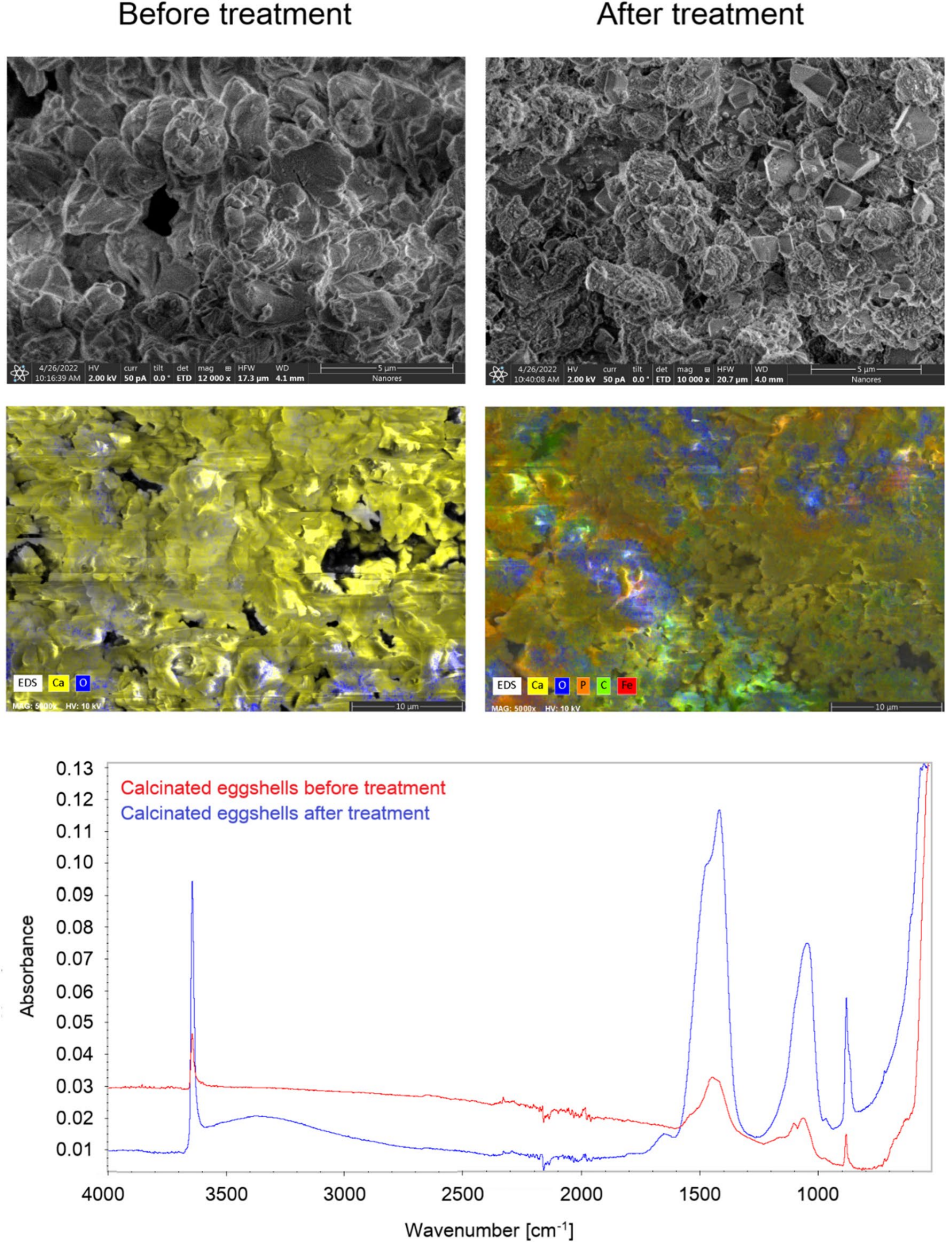
FT-IR spectra and SEM with EDS images of calcinated eggshells before and after sorption.

As in the case of dried eggshells, after wastewater treatment, the formation of small irregularities on the surface of the material can be observed on SEM images. EDS analysis showed the presence of sulphur and phosphorus as well as iron originating from industrial wastewater on the material surface, which also relates to the results obtained in the case of dried eggshells (Fig. 7). The change in surface area due to the calcination process was also confirmed in the values of the material's specific surface area. For dried eggshells surface area was only 0.15 m^2^/g, and pores volume 0.0012 cm^3^/g. For eggshells aster calcination these values were several times higher and were 4.15 m^2^/g and 0.0226 cm^3^/g, respectively.

Due to the calcination process, the calcined material consists mainly of calcium hydroxide. In addition, the use of calcinated eggshells in the treatment of acidic process effluent (initial pH about 2) causes an increase in pH to about 12–13. Taking this into account, it can be assumed that the calcinated eggshells’ mechanism of action is based on the precipitation occurring analogously to the use of pure hydroxide. This is also confirmed by similar results for calcinated eggshells, Ca(OH)_2_ and CaO, summarized in Table 4.

The material reuse tests conducted show that both dried eggshells and eggshells after calcination can be reused. Four cycles of removal of metal ions from industrial wastewater followed by material treatment using 0.1 M HCl, 0.1 M HNO_3_, 0.1 M NaOH and distilled water were performed. For eggshells after calcination, the sorption capacity was still 97% for all variants tested. For dried eggshells, the sorption capacity after 4 cycles was 95% for 3 of the solutions used, only slightly lower at 91% when using 0.1 M HNO_3_.

## Conclusion

In this paper, metal ion removal efficiency using materials of natural origin were investigated in terms of a real industrial effluent from a stainless steel electropolishing process. Not all materials selected for preliminary tests turned out to be applicable to real wastewater in terms of efficiency. Some of them, such as orange peel and algae, were not effective enough and absorbed large amounts of solution, making it difficult to separate the material from the wastewater solution after the treatment process.

The potential use of the plant material *Eclipta alba* was established. The best fits were obtained for the second order kinetic model and the Langmuir isotherm model. The rather not-very-high maximum sorption capacity of 8.64 mg/g obtained is due to the simultaneous occurrence of many different ions in the studied real wastewater, in addition to very acidic medium at which the sorption process was performed. Taking into account the removal efficiency of individual metal ions obtained at different initial concentrations of the effluent, it can be determined that iron and chromium are the easiest to remove from the solution. Nickel and copper are more difficult to remove. This is a characteristic order for this type of wastewater, which was also noted by authors when using peat as a sorbent in previous work.

On the basis of FT-IR investigations and comparison of the spectra obtained, it was determined that dried eggshells are very close to CaCO_3_, as much as calcined eggshells are to Ca(OH)_2_. Taking into account the presence of mainly calcium hydroxide in eggshells after calcination, and with the pH of the waste water solution after the treatment process being about 12–13, the calcinated eggshells’ mechanism of action resulting mainly from the precipitation of metal hydroxides from the solution was determined. In the case of dried eggshells containing mainly calcium carbonate in their composition, the mechanism has not been conclusively resolved. It can be complex and may involve precipitation, adsorption on the dried eggshells’ surface, as well as ion exchange.

Both dried and calcinated eggshells showed high metal ions removal efficiencies of 95.3–97.6% and 97.4–99.9%, respectively. The highest reductions were achieved for Fe(III) where virtually 100% removal was achieved in all cases, and for Cr(III) where the values were slightly lower but still exceeded 90%. More sizable differences can be observed for Ni(II) and Cu(II). The character of dried eggshells as being similar to calcium carbonate, with that of calcinated eggshells being similar to calcium hydroxide, both determined on the basis of FT-IR studies, were also confirmed by the results in terms of the effectiveness of these materials on the studied industrial wastewater.

## Data Availability

All data generated or analysed during this study are included in this published article.
